# Normal Ribosomal Biogenesis but Shortened Protein Synthetic Response to Acute Eccentric Resistance Exercise in Old Skeletal Muscle

**DOI:** 10.3389/fphys.2018.01915

**Published:** 2019-01-14

**Authors:** Daniel W. D. West, George R. Marcotte, Courtney M. Chason, Natalie Juo, Leslie M. Baehr, Sue C. Bodine, Keith Baar

**Affiliations:** ^1^Department of Physiology and Membrane Biology, University of California, Davis, Davis, CA, United States; ^2^Department of Neurobiology, Physiology and Behavior, University of California, Davis, Davis, CA, United States; ^3^VA Northern California Health Care System, Mather, CA, United States

**Keywords:** ribosome biogenesis, ER stress, ubiquitin proteasome, IRS-1 signaling, anabolic resistance, sarcopenia

## Abstract

Anabolic resistance to feeding in aged muscle is well-characterized; however, whether old skeletal muscle is intrinsically resistant to acute mechanical loading is less clear. The aim of this study was to determine the impact of aging on muscle protein synthesis (MPS), ribosome biogenesis, and protein breakdown in skeletal muscle following a single bout of resistance exercise. Adult male F344/BN rats aged 10 (Adult) and 30 (Old) months underwent unilateral maximal eccentric contractions of the hindlimb. Precursor rRNA increased early post-exercise (6–18 h), preceding elevations in ribosomal mass at 48 h in Adult and Old; there were no age-related differences in these responses. MPS increased early post-exercise in both Adult and Old; however, at 48 h of recovery, MPS returned to baseline in Old but not Adult. This abbreviated protein synthesis response in Old was associated with decreased levels of IRS1 protein and increased BiP, CHOP and eIF2α levels. Other than these responses, anabolic signaling was similar in Adult and Old muscle in the acute recovery phase. Basal proteasome activity was lower in Old, and resistance exercise did not increase the activity of either the ATP-dependent or independent proteasome, or autophagy (Cathepsin L activity) in either Adult or Old muscle. We conclude that MPS and ribosome biogenesis in response to maximal resistance exercise in old skeletal muscle are initially intact; however, the MPS response is abbreviated in Old, which may be the result of ER stress and/or blunted exercise-induced potentiation of the MPS response to feeding.

## Introduction

Skeletal muscle anabolic resistance is a term that describes a reduced anabolic response to a given stimulus (for example: feeding, resistance exercise, or chronic loading), and is often used in an age-related context – i.e., old individuals exhibit anabolic resistance compared with young (Cuthbertson et al., 2005). Further, age-related loss in muscle mass appears to be underpinned by reduced responses to acute anabolic stimuli (Volpi et al., 2000) as opposed to lower basal rates of MPS (Volpi et al., 2001).

Age-related anabolic resistance to dietary protein feeding is relatively well-characterized (Volpi et al., 2000; Cuthbertson et al., 2005; Katsanos et al., 2005; Moore et al., 2015); by contrast, the effects of aging on the anabolic response to mechanical loading are poorly understood (Drummond et al., 2012; Baehr et al., 2016; Brook et al., 2016). Blunted rates of MPS and mTORC1 signaling in old individuals have been reported after moderate-to-high intensity resistance exercise (Kumar et al., 2009, 2012; Fry et al., 2011). However, observations of anabolic resistance to exercise may be confounded by underlying age-related differences in baseline activity/exercise habits (i.e., “active” old vs. sedentary old) (Burd et al., 2012b) and/or differences in motor unit activation during the exercise bout. Old individuals are reported to exhibit a reduced ability to drive their motor units at high muscle contraction intensities (Kamen et al., 1995). Thus, it is unclear from human studies the degree to which sarcopenia is the result of intrinsic and/or extrinsic factors – i.e., the contribution of the aging process *per se* vs. age-related reductions in habitual physical activity or motor unit activation (Clarke, 2004).

In well-controlled animal studies, age-related impairments in ribosome biogenesis and hypertrophy occur in response to synergist ablation (Hwee and Bodine, 2009; Kirby et al., 2015); however, synergist ablation increases muscle mass at a rate of 15–30% per week, whereas resistance exercise-induced gains are approximately 1–2% per week (Baar and Esser, 1999; Wernbom et al., 2007). Therefore, even though extreme models of muscle loading show impairments in ribosome biogenesis and hypertrophy with age, whether the response to physiological loading is impaired is unclear. In this study, we addressed this gap using a single bout of a well-established animal model of eccentrically biased resistance exercise (Wong and Booth, 1990; Baar and Esser, 1999; Chen et al., 2002; Hamilton et al., 2010; West et al., 2016). A detailed evaluation of this and other skeletal muscle hypertrophy models is beyond the scope of this paper; for more discussion on this topic readers are referred to reviews elsewhere (Timson, 1990; Cholewa et al., 2014).

The anabolic response to resistance exercise is characterized by alterations in skeletal muscle translational activity and capacity (Henshaw et al., 1971; Stec et al., 2015; West et al., 2016). Therefore, the primary purpose of the present study was to determine whether exercise-induced increases in translational activity and capacity are impacted by age after an acute bout of eccentric exercise using a model of maximal motor unit activation. To achieve this aim, we determined MPS, ribosome biogenesis, and acute mTORC1 signaling in recovery (6, 18, 48 h) from maximal resistance exercise in adult and old animals.

Even though much of the focus for declining muscle mass has concentrated on the protein synthesis side of the equation, age-related declines in autophagy and proteasome function may reduce clearance of dysfunctional proteins and contribute to cellular senescence (Anvar et al., 2011; Lopez-Otin et al., 2013; Hwee et al., 2014; Hentila et al., 2018). A senescent cell phenotype in skeletal muscle may impact the growth response to anabolic stimuli. Conversely, in the compensatory hypertrophy model, large increases in ubiquitin proteasome pathway (UPP) activity accompany increases in MPS and muscle mass (Baehr et al., 2014). These data suggest that increased proteasome activity is needed for chronic growth. Additionally, MuRF1 (an E3 ligase in the UPP) knockout animals exhibit elevated proteasome activity throughout their lifespan and this was associated with protection against age-related loss of muscle mass as well as improved growth in response to overload (Hwee et al., 2014). Taken together, proteasome function may be decreased in aging skeletal muscle and this may contribute to the impaired response to anabolic stimuli. Therefore, a second aim of this study was to investigate age-related differences in protein degradation pathways after resistance exercise.

## Materials and Methods

### Animals and Exercise Protocol

Adult (10 months) and old (30 months) male Fischer 344-Brown Norway rats (Table 1) were used according to a protocol approved by the University of California Davis Animal Care and Use Committee. Animals were anesthetized (isoflurane inhalation, 2.5%) before undergoing acute unilateral electrical stimulation of the sciatic nerve to activate the hindlimb muscles. In this model of resistance exercise, the muscles in the anterior compartment (tibialis anterior; extensor digitorum longus) undergo high-force lengthening contractions as a result of the stronger antagonist muscles in the posterior compartment (gastrocnemius; plantaris, soleus) (Wong and Booth, 1990). After stimulation, animals were placed back in their cages and allowed *ad libitum* access to food. Thirty minutes before muscle collections, animals were administered puromycin (0.02 μmol g^−1^ body weight by I.P. injection) for the determination of protein synthesis via the surface-sensing of translation method (Goodman et al., 2011). Animals were then anesthetized and hindlimb muscles were surgically removed and frozen in liquid nitrogen 6, 18, or 48 h following stimulation. The tibialis anterior muscle, which is a predominantly fast-twitch muscle, was powdered with a liquid nitrogen-cooled mortar and pestle before further analysis.

**Table 1:** Baseline body weight, hindlimb muscle weights and total RNA concentration in adult and old.

	Adult (10 months)	Old (30 months)	Old relative to adult
Body weight (g)	447.1	544.9	+21.9% *P* < 0.001
Muscle weight (g)			
TA	0.812 ± 0.054	0.712 ± 0.063	−12.4% *P* = 0.001
EDL	0.195 ± 0.011	0.181 ± 0.010	−7.2% *P* = 0.009
SOL	0.199 ± 0.019	0.179 ± 0.016	−9.9% *P* = 0.021
PLN	0.438 ± 0.034	0.365 ± 0.026	−16.7% *P* < 0.001
Total RNA (μg RNA/mg wet tissue)	0.800 ± 0.028	0.857 ± 0.057	+7.1% *P* < 0.001

*Values are means ± SD. TA, tibialis anterior; EDL, extensor digitorum longus; SOL, soleus; PLN, plantaris.*

### Protein Levels

**FIGURE 1:**
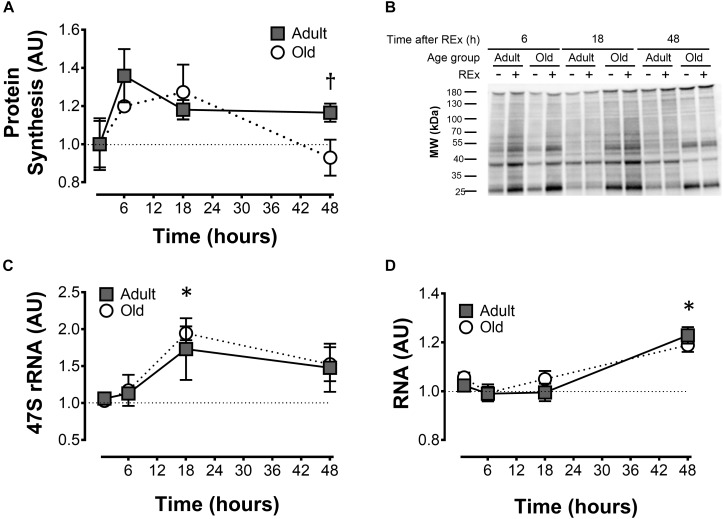
Muscle protein synthesis and ribosome biogenesis after acute unilateral resistance exercise in adult (10 months) and old (30 months) rat skeletal muscle. Muscle protein synthesis **(A)** with representative blot showing puromycin incorporation **(B)**; 47S rRNA **(C)**, and total RNA **(D)**. ^†^Difference between Adult and Old at the same time point, *P* < 0.05. ^∗^Main effect of time; different from 0 to 6 h, *P* < 0.05. Values are expressed as experimental/contralateral control muscles, means ± SEM; *n* = 6/group.

Levels of select proteins, as well as puromycin integration, were measured by western blot. Briefly, an aliquot of frozen tissue powder was homogenized in a sucrose lysis buffer (50 mM Tris pH 7.5, 250 mM sucrose, 1 mM EDTA, 1 mM EGTA, 1% Triton X100, 50 mM NaF, 1 mM NaVO4 Na_2_(PO_4_)_2_, and 0.1% DTT), centrifuged (10,000 *g* × 10 min), and the supernatant collected for protein concentration measurement (DC protein assay, Bio-Rad, cat. 500-0116). Protein concentrations were equilibrated, combined with Laemmli sample buffer, and heated at 95°C for 5 min. Gradient polyacrylamide gels (4–20%) containing non-exercised and exercised samples in adjacent wells were used to separate proteins by electrophoresis (200 V for 45 min), transferred to nitrocellulose membrane (wet transfer, 100 V for 1 h), blocked in 1% fish skin gelatin, and washed in Tris-buffered saline 0.1% tween (TBST) before overnight incubation in primary antibody (1:1000 in TBST) at 4°C. Primary antibodies were from: Millipore – puromycin (cat. MABE343); Cell Signaling Technologies (Danvers, MA, United States) – BiP (cat. 3183), CHOP (cat. 5554), phospho-eEF2 Thr56 (cat. 2331), phospho-eIF2α Ser51 (cat. 9721S), IRS1 (cat. 2382S), phospho-rpS6 Ser240/244 (cat.2215), phospho-S6K1 Thr389 (cat. 9205); and Santa Cruz Biotechnology (Santa Cruz, CA, United States) – phospho-UBF Ser637 (cat. 21639). Membranes were incubated in secondary antibody (1:10,000 in TBST; 1 h at RT) before protein expression detection by chemiluminescence (Millipore, cat. WBKLS0500). Blots were normalized for protein loading via Ponceau stain (Figure 1B). Images for densitometry analysis were captured with a Bio-Rad ChemiDoc MP imaging system and quantified using Image Lab Software (Bio-Rad, v. 5).

### Total RNA and Gene Expression

Total RNA was extracted, from a pre-weighed aliquot of frozen TA muscle powder, using RNAzol RT (Sigma, cat. R4533) according to manufacturer’s instructions. Absorbance was quantified by spectrophotometry (Epoch Microplate Spectrophotometer, BioTek Instruments Inc.). One microgram of RNA was converted to cDNA using a reverse transcription kit (Life Technologies, cat. 4368814) according to manufacturer’s instructions. cDNA was diluted 10-fold before analysis by quantitative RT-PCR. Gene expression was calculated using the delta delta threshold cycle method (Livak and Schmittgen, 2001) (experimental vs. contralateral control) and GAPDH was used as a housekeeping gene. There was no difference in the absolute C_T_ of GAPDH as a result of either age (Adult = 15.104 +/− 0.410; Old = 15.192 +/− 0.381) or exercise (Adult = 15.219 +/− 0.407; Old = 15.110 +/− 0.320). Primer sequences are shown in Table 2.

**Table 2:** 5′ to 3′ sequences of primers used for quantitative RT-PCR.

Gene	Forward	Reverse
**Rat**		
c-Myc	CAGCAGCGACTCT GAAGAAGAAC	GATGACCCTGA CTCGGACCTC
Glyceraldehyde 3-phosphate dehydrogenase	GTCATCCCA GAGCTGAACGG	GCCTGCTTC ACCACCTTCT
Internal transcribed spacer 1	TCCGTTTTCT CGCTCTTCCC	CCGGAGAGAT CACGTACCAC
Nucleolin	AAAGTGCCCC AGAACCCACA	TGGCTGACTTC TCGCATTAGG
Nucleophosmin	TGTCCAGGTTC AATTGCCAAG	CCAAGTAAAG GGCGGAGTT
TATA box binding protein-assoc. factor RNA Pol I B	CATCTTTGCTGT CGAGTCTTGG	GGATGGAGGTA GCAGTCTTCAG

### Proteasome and Cathepsin L Activity Assays

Proteasome and cathepsin L activity assays were performed as previously described (Gomes et al., 2012). Briefly, frozen muscle powder was dounce-homogenized in cold buffer (50 mM Tris pH 7.5, 1 mM EDTA, 150 mM NaCl, 5 mM MgCl_2_, 0.5 mM DTT) before centrifugation (12,000 g × 30 min at 4°C) and collection of the supernatant. Supernatant protein concentrations were determined and equal protein quantities were assayed: 8–20 μg/well for proteasomal subunit assays, and 34 μg/well for cathepsin L assays. Samples from REx and contralateral control legs, as well as adult and old groups, were run on the same plate. Fluorescence of tagged substrates (proteasome: 20–100 μM Leu-Leu-Val-Tyr—4-amino-7-methyl coumarin, BACHEM, cat. I–1395; cathepsin L, Z-Phe-Arg-MCA, Peptide Instituted Inc, Code: 3095-v) was measured kinetically (Fluoroskan Ascent 2.5, Thermo Electron, Waltham, MA, United States) to ensure assay linearity. Activity was determined by calculating the difference between wells with and without inhibitor (proteasome: Bortezomib, 2–10 μM; Calbiochem cat. 504314; cathepsin L: cathepsin L inhibitor I: 10 μM Calbiochem, cat. 219421).

### Statistical Analysis

Two-way ANOVAs were used to analyse time course data (age × time) and proteasome data (age × condition) with Tukey’s *post hoc*. Factors (levels) were as follows: age (Adult vs. Old), time (6, 18, and 48 h), and condition (experimental vs. control leg). Data was log transformed where appropriate to correct skewness and unequal variance before statistical analyses. Statistical analyses were performed using SigmaStat software (v.3.1, Systat Software, San Jose, CA, United States); statistical significance was set at *P* < 0.05.

## Results

### Muscle Protein Synthesis and Ribosome Biogenesis

Muscle protein synthesis was moderately elevated (18–33%) after resistance exercise, and this response was more sustained in adult animals (Adult > Old at 48 h, *P* = 0.046; Figure 1A). Precursor ribosomal RNA expression was elevated at 18 h compared with 6 h (*P* < 0.05) and by 48 h precursor rRNA was in decline but not yet back to baseline (Figure 1C). Total RNA was elevated in Adult (23%, experimental/contralateral control) and Old (19%) at 48 h after exercise (Figure 1D), but was not different between age groups (*P* = 0.38). Total RNA at 48 h was elevated compared with 6 and 18 h after exercise (main effect of time, both *P* < 0.001). In control legs, total RNA concentration (μg RNA/mg wet tissue) was modestly (+7% in Old) but significantly (*P* < 0.001) higher in Old compared with Adult (Table 1). Hindlimb muscle weights of the 10 and 30 month old animals are shown in Table 1.

### mTORC1 Signaling

**FIGURE 2:**
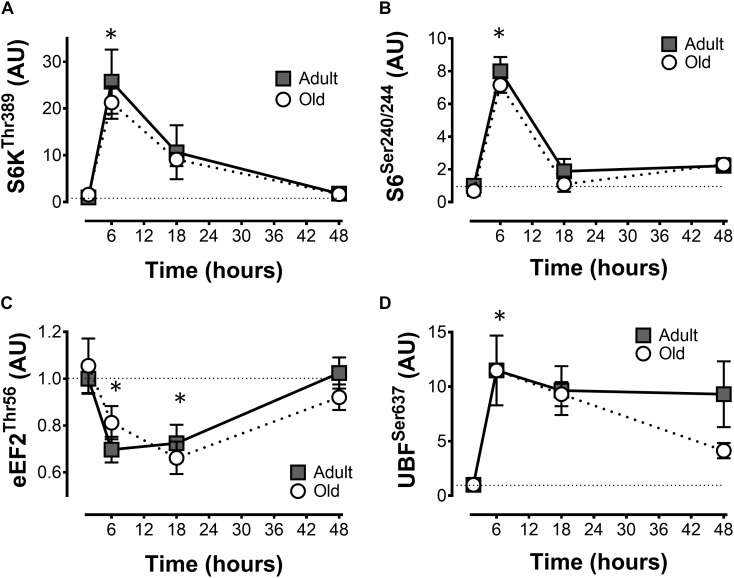
mTORC1 signaling after acute unilateral resistance exercise in adult (10 months) and old (30 months) rat skeletal muscle. Ribosomal S6 kinase **(A)**, ribosomal protein S6 **(B)**, eukaryotic elongation factor 2 **(C)**, and upstream binding factor **(D)** phospho-protein levels. ^∗^Main effect of time; different from non-stimulated control group, *P* < 0.05. Values are expressed as experimental/contralateral control muscles, means ± SEM; *n* = 6/group.

The phosphorylation of S6K1^*Thr*389^ and ribosomal protein S6^*Ser*240/244^ in response to exercise peaked at 6 h, declined at 18 h and returned to baseline levels at 48 h (Figures 2A,B). Elongation factor 2^*Thr*56^ phosphorylation decreased (Figure 2C), and UBF^*Ser*637^ (Figure 2D) phosphorylation increased following exercise. All of these responses were similar in Adult and Old muscles across the recovery time course.

### Gene Expression

**FIGURE 3:**
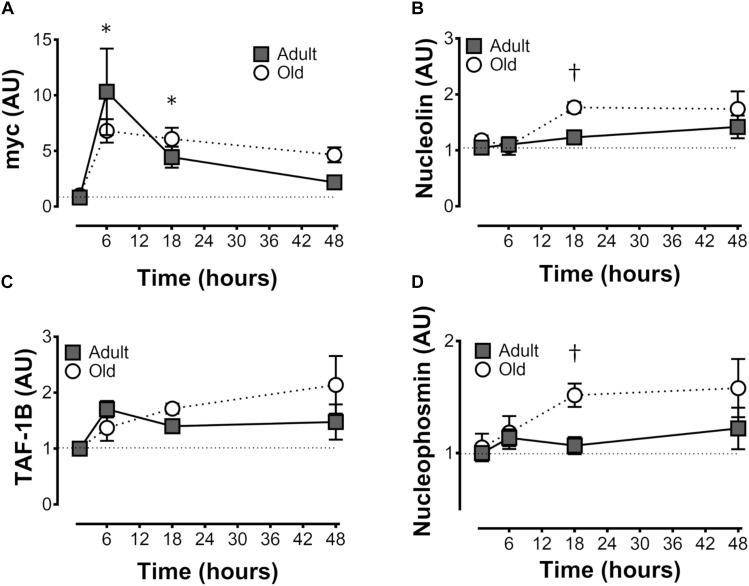
Expression of genes associated with ribosome biogenesis after unilateral resistance exercise in adult (10 months) and old (30 months) rat skeletal muscle. c-Myc **(A)**, nucleolin **(B)**, TATA box binding protein-associated factor RNA Pol I B (TAF1B; **C**), and nucleophosmin **(D)** mRNA abundance. ^†^Difference between Adult and Old at the same time point, *P* < 0.05. Values (means ± SEM) are expressed relative to GAPDH and the contralateral control using the delta-delta Ct method; *n* = 6/group.

c-Myc expression was elevated by exercise in Adult and Old at 6 and 18 h, with no effect of age (Figure 3A). Nucleolin and nucleophosmin, myc target genes that play a role in precursor rRNA splicing, showed very similar (to each other) patterns of expression, and were both greater at 18 h of recovery in Old vs. Adult (*P* = 0.034 and 0.025 for nucleolin and nuclophosmin, respectively, Figures 3B,D). TATA box binding protein-associated factor RNA Pol I B (TAF1B), a component of the pre-initiation complex that mediates Pol I transcription of rDNA, tended to gradually rise to a “peak” at 48 h in Old, whereas TAF1B expression peaked at 6 h in Adult before declining at later time points (*P* = 0.056 for age × time interaction; Figure 3C).

### ER Stress

**FIGURE 4:**
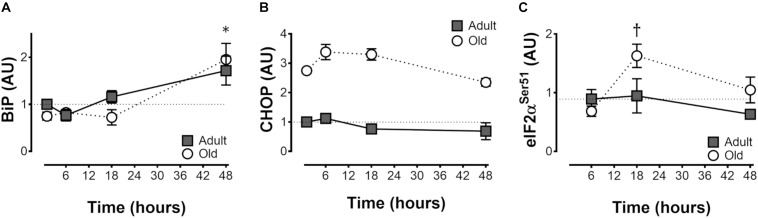
ER stress protein levels after acute unilateral resistance exercise in adult (10 months) and old (30 months) rat skeletal muscle. Endoplasmic reticulum binding protein (BiP; **A**), CCAAT-enhancer-binding protein homologous protein (CHOP; **B**), and phospho eukaryotic initation factor 2α (eIF2α; **C**). ^∗^Main effect of time; different from non-stimulated control group, *P* < 0.05. Main effect of age for CHOP, *P* < 0.001 **(B)**. ^†^Difference between Adult and Old at the same time point, *P* < 0.05. *n* = 6/group.

Protein levels of the adaptive BiP were elevated by resistance exercise at 48 h in both Adult and Old (time effect, *P* < 0.001; Figure 4A), whereas CHOP was unchanged by resistance exercise but was constitutively higher in Old (*P* < 0.001; Figure 4B). Phospho-eIF2α Ser51 was elevated by resistance exercise at 18 h in Old but not Adult (Figure 4C).

### Insulin Signaling

**FIGURE 5:**
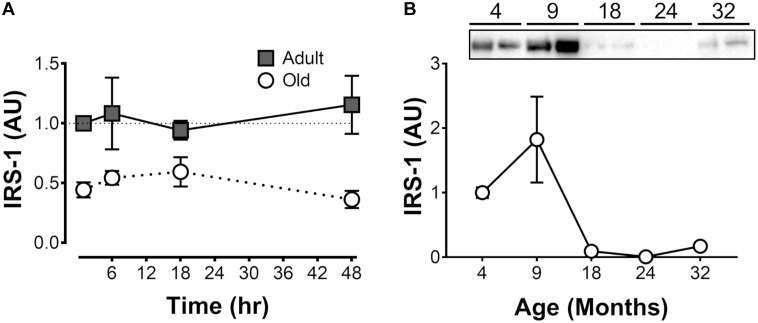
Insulin receptor subunit 1 (IRS-1) protein levels (i) after acute unilateral resistance exercise in adult (10 months) and old (30 months) rat skeletal muscle (**A**; *n* = 6/group), and (ii) across the lifespan **(B)**. Tissue for samples in panel **B** is from the National Institute on Aging Aged Rodent Tissue Bank.

We hypothesized that the premature return to baseline in protein synthesis rates may be related to the inability to potentiate the acute effect of feeding. Since insulin signaling is required for the acute response to feeding, we determined IRS-1 levels in the Adult and Old muscles. Consistent with an early return to basal protein synthesis, IRS-1 levels were ∼50–70% lower in Old vs. Adult and were unaffected by exercise (Figure 5A). To determine whether the decrease in IRS-1 was a function of age in this rat model, we analyzed quadriceps muscles from 4, 9, 18, 24, and 32-month old F344/BN rats obtained from the NIA tissue repository. Consistent with our Adult and Old groups, levels of IRS-1 in muscle drop more than 10-fold between 4 and 24 months old in the samples from the NIA tissue repository (Figure 5B).

### Proteasome and Cathepsin L Activity

**FIGURE 6:**
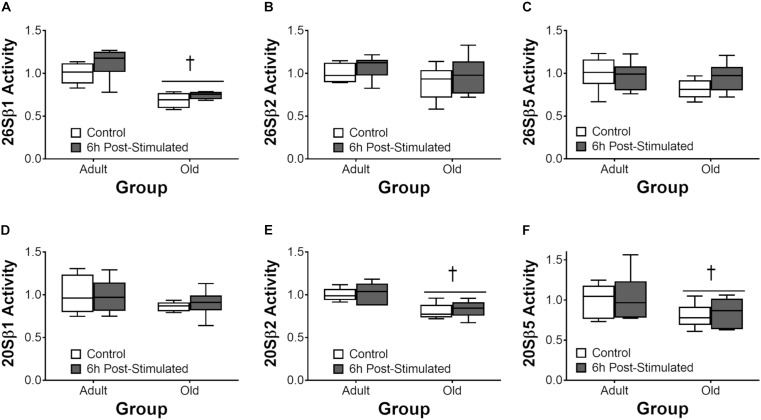
ATP-dependent (26S, **A–C**) and ATP-independent (20S, **D–F**) proteasomal subunit activities 6 h after acute unilateral resistance exercise in adult (10 months) and old (30 months) rat skeletal muscle. Boxes represent 25th to 75th percentiles, horizontal lines within boxes represent medians, and whiskers represent minimums and maximums. *n* = 6/group. ^†^Main effect of age, *P* < 0.05.

**FIGURE 7:**
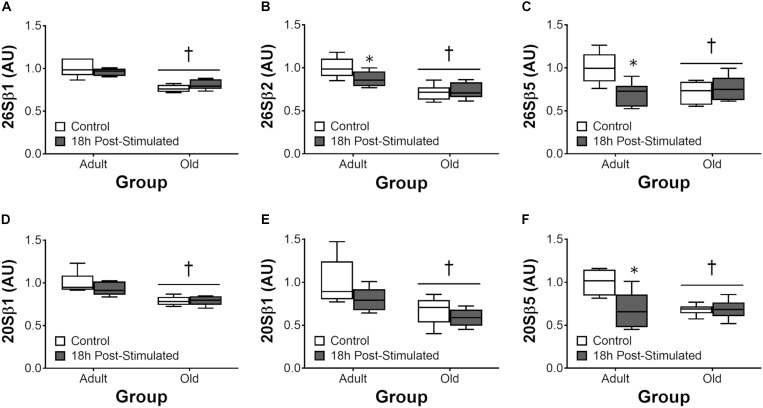
ATP-dependent (26S, **A–C**) and ATP-independent (20S, **D–F**) proteasomal subunit activities 18 h after acute unilateral resistance exercise in adult (10 months) and old rat skeletal muscle. Boxes represent 25th to 75th percentiles, horizontal lines within boxes represent medians, and whiskers represent minimums and maximums. *n* = 6/group. ^†^Main effect of age, *P* < 0.05. ^∗^Difference between exercised and contralateral control muscle within age group.

**FIGURE 8:**
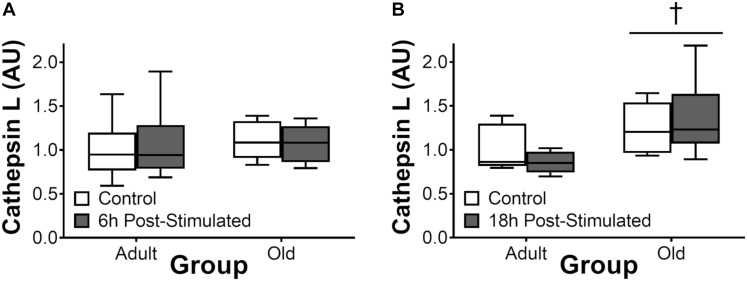
Cathepsin L activity 6 h **(A)** and 18 h **(B)** after acute unilateral resistance exercise in adult (10 months) and old (30 months) rat skeletal muscle. Boxes represent 25th to 75th percentiles, horizontal lines within boxes represent medians, and whiskers represent minimums and maximums. *n* = 6/group. ^†^Main effect of age, *P* < 0.05.

Proteasome activity was not different from control at 6 h after acute exercise in Adult or Old (Figure 6). Eighteen hours after exercise, the activity of the 26S β2, 26S β5, and 20S β5 proteasomal subunits decreased (all *P* < 0.05) in Adult (Figure 7). Interestingly, proteasome activity was generally lower in Old vs. Adult (6h = 26S β1, and 20S β2 and β5, main effect of age, all *P* < 0.05; 18 h = 26S β1, β2, β5, and 20S β1, β2, β5, main effect of age, all *P* < 0.05). Cathepsin L activity was greater in Old at the 18 h time point (age effect, *P* = 0.008), but was unchanged by exercise (both age groups, at 6 and 18 h of recovery; Figure 8).

## Discussion

The present study made age-based comparisons of MPS, ribosome biogenesis, mTORC1 signaling, ER stress, components of insulin signaling and protein degradation after acute eccentric exercise. The major findings are as follows. Precursor ribosomal RNA and total RNA increased similarly between age-groups after resistance exercise. Increases in MPS at 6 and 18 h after exercise were similar between Adult and Old; however, MPS was significantly greater in Adult than Old late in recovery (48 h). mTORC1 signaling was similar in Adult and Old after resistance exercise; however, levels of IRS-1 were significantly lower in Old animals. ER stress was higher- and proteasome activity was lower- at baseline in Old vs. Adult, but neither was induced by exercise.

We previously observed a trend toward increased total RNA 36 h after the same acute resistance exercise protocol used in the present study (West et al., 2016). Here, precursor ribosomal RNA abundance increased at 18 h of recovery and this preceded a significant increase in total RNA at 48 h. These data suggest that exercise-induced enhancement of ribosome biogenesis results in a significant increase in total RNA by 48 h in this model. These data are consistent with the acute stimulation of ribosome biogenesis in humans following resistance exercise (Kim et al., 2007). Consistent with our previous observation (West et al., 2016), the expression of nucleolin and nucleophosmin genes returned to baseline levels by 18 h in the Adult group; conversely, in Old, the expression of these myc target genes remained elevated at 18 h, implying a slower/more prolonged time course of exercise-mediated induction of these genes. Though the dynamics of the gene responses differed slightly, both Adult and Old were able to increase total RNA at 48 h. Taken together, both adult and old animals appear capable of inducing ribosome biogenesis in response to acute muscle contraction and this similar induction does not explain the age-based differential MPS response.

The finding of apparently normal ribosome biogenesis (at least insofar as rRNA accumulation) after an acute bout of resistance exercise is in contrast to the impaired ribosome biogenesis reported with overload in old mice (Kirby et al., 2015) and resistance training in elderly humans (Stec et al., 2015). One explanation is that old muscle activates ribosome biogenesis in response to an acute exercise stimulus, but that chronic loading results in an impaired response. Future training studies using the present exercise model would be valuable to clarify this hypothesis; in advance, support for this theory comes from work showing that old rats exhibit more metabolic stress with overload and this results in less hypertrophy than young controls (Thomson and Gordon, 2005). Here, our data suggests that aging does not impair the initial accumulation of total RNA, of which ∼85% is ribosomal RNA (Zak et al., 1967), in response to an acute bout of exercise. Ribosome biogenesis occurs predominately in the nucleolus, a process we recently visualized in muscle cells *in vitro* (West et al., 2016). Interestingly, the nucleolus is becoming increasingly recognized as a site for sensing cell stress (Olson, 2004; Nakamura and Kimura, 2017). Thus, future work using ribosome function assays at later time points, and/or in response to repeated stimuli or metabolic stress, would be beneficial to determine whether translational capacity is compromised in old muscle as a result of elevated cellular stress.

The possibility of differences in ribosome assembly/function aside, ribosomal mass accrual appeared to be normal at 48 h post-exercise in Old; in contrast, the protein synthesis response was not; with MPS returning to baseline in Old but not Adult at 48 h. While the specific mechanism for the abbreviated MPS response is unclear, several possibilities exist. First, we have shown that metabolic and ER stress associated with aging (Baehr et al., 2016) can act as a molecular brake on anabolic signaling in skeletal muscle (Hamilton et al., 2014). Whereas the mechanical stimulus to activate mTORC1 signaling did not appear to be intrinsically impaired in Old (S6K, rpS6, and eEF2 were all activated similarly between Adult and Old), it is possible that age-related ER stress may limit protein synthesis. CHOP was higher at all time points in Old, and both BiP and CHOP were elevated at 48 h in Old. Phosphorylated levels of eIF2α were elevated by resistance exercise at 18 h in Old but not Adult. Ser51 phosphorylation of the eIF2 alpha subunit blocks the GEF activity of eIF2B, reducing availability of eIF2-GTP-Met-tRNA_i_ and inhibiting the global rate of translation initiation (Donnelly et al., 2013; Kashiwagi et al., 2017). Our data showing increased levels of phospho-eIF2α that precede increased BiP, suggests that BiP may not be the trigger to initiate eIF2α phosphorylation as has been proposed (Cui et al., 2011), or at least not in old skeletal muscle in post-exercise recovery. Altogether, the cellular environment in old skeletal muscle, characterized by high CHOP, may down-regulate protein synthesis, which is initially stimulated by intact mTORC1 signaling in Old.

A second potential mechanism to explain the accelerated return to baseline MPS in Old muscle post-exercise is decreased exercise-induced sensitization to feeding (Burd et al., 2011). The early MPS response is driven by the loading stimulus and is independent of insulin/IGF-1 receptor signaling (Spangenburg et al., 2008; Jacobs et al., 2017). This mechanical response appears to be intact in Old since we observed no anabolic resistance in the activation of mTORC1. However, at 48 h, it is possible that the prolonged increase in MPS is mediated through the potentiation of the feeding response. Unlike the exercise-induced activation of mTORC1 and protein synthesis, the feeding response requires insulin/IGF-1 signaling (Spangenburg et al., 2008; Hamilton et al., 2010; Jacobs et al., 2017). Muscle from both our 30 month old animals and samples from the NIA tissue bank showed that total IRS1 protein content is significantly lower in Old muscle. Since IRS-1 is the first protein recruited to the insulin receptor in response to hormonal activation, it is required for the insulin-dependent activation of PI3-kinase/Akt/mTORC1 and protein synthesis in muscle but not for the mechanical activation of mTORC1 (Hornberger and Chien, 2006). In the NIA tissue bank samples, IRS-1 levels peaked at 9 months and then there was a sharp decline in IRS-1 protein with age. We note that while we have discussed ER stress- and IRS-1-based mechanisms separately above there is evidence to suggest that ER stress inhibits IRS-1 signaling in skeletal muscle as well (Koh et al., 2013). Thus, we speculate that a relative absence of IRS-1 in old muscle – and perhaps reduced activity of remaining IRS-1 – may contribute to a lack of exercise-induced feeding potentiation of MPS at late time points (e.g., 48 h) following resistance exercise.

The feeding-mediated potentiation of human exercise-induced rates of synthesis occurs in the myofibrillar and not the sarcoplasmic protein pool (Burd et al., 2011) and thus, if blunted with aging, may contribute to sarcopenia. Sarcopenia, which is the result of chronically negative net protein balance, is somewhat paradoxical. Our finding of no apparent deficits in translation capacity (on the basis of greater total RNA) add to previous work showing that basal MPS is similar (Volpi et al., 2001) or higher (Smith et al., 2012) in old vs. adult muscles, despite reduced muscle mass (7–17% in the present study; Table 1). In non-exercised control muscle, ribosomal density was modestly (+7%) but significantly higher in Old compared with Adult. While there are plausible hypotheses (e.g., increased ribosomal density to compensate for deficits in ribosome function/translational efficiency) – in short, we do not know the mechanism(s) underpinning this observation but note that similar or greater ribosome densities have been reported by others when comparing old vs. young (Haddad and Adams, 2006). Thus, our data indirectly support the notion that sarcopenia is underpinned by recurrent episodes of anabolic resistance.

Our work also supports a mechanism for age-related anabolic dysfunction that is a result of age-related differences in muscle damage. Using the same model, we have previously shown that there is greater membrane damage in old animals in response to exercise due to impaired lateral force transmission (Hughes et al., 2017). Further, supporting evidence shows that old individuals suffer greater contraction-induced muscle injury in response to acute unaccustomed loading (Ploutz-Snyder et al., 2001). The result of this may be that, in the present study, both Adult and Old were able to increase protein synthesis after the exercise bout, but the proteins synthesized in Adult were primarily the myofibrillar proteins involved in muscle growth, whereas in Old more protein synthesis was directed toward injury repair. The surface sensing of translation (SUnSET) method does not characterize which proteins are synthesized following exercise; alternative techniques (e.g., isotopic labeling) that can distinguish which proteins – on a subfraction (Burd et al., 2011) or individual protein (Hines et al., 2015) basis – are being synthesized would enhance our understanding of the acute response to resistance exercise in Adult and Old muscles. From our data, we hypothesize that the impact of aging on myofibrillar protein accretion is two-fold: (1) impaired nutrient signaling decreases the feeding-mediated potentiation of exercise-induced myofibrillar protein synthesis, and (2) relative to adult muscle, protein synthesis in old muscle may be directed toward repairing muscle damage rather than synthesizing the myofibrillar proteins that will increase muscle size and strength.

The UPP is the major protein degradation pathway in skeletal muscle (Rock et al., 1994). Our data showing reduced activity of the UPP in Old at baseline is paradoxical insofar as the net protein balance equation and is in contrast to previous reports (Hepple et al., 2008; Altun et al., 2010). Nevertheless, our data is consistent with the findings of others (Anvar et al., 2011; Hwee et al., 2014), leading us to hypothesize that age-related reductions – not elevations – in skeletal muscle proteasome activity contribute to cellular dysfunction which may in turn contribute to the gradual pathological phenotype characterized by sarcopenia, as has been suggested to occur with diabetes (Al-Khalili et al., 2014). The decrease in proteasome function that we observed in Old may have contributed to the higher ER stress levels (via reduced ER-associated degradation) we observe in old skeletal muscle, here and previously (Baehr et al., 2016). In the context of exercise, the present findings are in agreement with our previous observation (West et al., 2016) of no induction of proteasome activity following acute resistance exercise. Likewise, cathepsin L activity was not significantly altered by resistance exercise, and was either no different (6 h post-exercise) or elevated (18 h) in Old vs. Adult overall. Thus, collectively, we do not detect significant exercise-induced increases in protein degradation by the proteasome or autophagy systems, but suspect that age-related impairments in the basal activity of these pathways contribute to age-related impairments in myofiber remodeling and adaptation to loading.

### Limitations

We acknowledge that the animal model used presents several limitations insofar as the generalizability of our findings to the geriatric population. First, the tibialis anterior muscle that was examined is comprised of a high proportion of fast-twitch fibers, higher than human ambulatory muscles. Accordingly, given the impact of fiber type on protein metabolism (Dickinson et al., 2010; Goodman et al., 2012.), it is unclear whether the same findings would be observed in the more oxidative/“mixed” fiber-types seen in people. Examining the impact of aging on outcomes presented herein in muscles of a different fiber type composition (e.g., soleus) would be an interesting avenue for future work. Second, we intentionally used a model of maximal motor unit activation to address the issue of whether or not age-related differences in motor unit activation (Kamen et al., 1995) contribute to age-related anabolic resistance to resistance exercise. This was a mechanisms-targeted strategy to remove motor unit activity as a variable and we acknowledge that this exercise paradigm does not mimic the exercise patterns of the geriatric population. Finally, because the electrically stimulated eccentric contractions used in the present study do not mimic the general exercise patterns of geriatric populations, our findings should not be applied indiscriminately to any such population undertaking resistance exercise. Having said that, researchers contend that electrical stimulation is an effective strategy to attenuate muscle loss in elderly and clinical populations (Wall et al., 2012), and that eccentric exercise is safe, feasible, and relevant even in clinical populations (LaStayo et al., 2014). Further, work showing that when lifting a weight to concentric failure humans recruit all of the motor units within the active muscle (Burd et al., 2012a), suggesting that older individuals can benefit from these results by lifting a weight to failure. Thus, while our research model does not represent the general exercise patterns of elderly, the model remains within the bounds of a physiologically relevant loading stimulus while yielding mechanistic insight into the response to exercise in young vs. old individuals.

## Conclusion

In conclusion, our data suggest that when motor units are fully recruited to undergo maximal eccentric loading, the capacity of old muscle to elevate MPS in response to an acute bout of exercise is initially intact but the duration of the response is truncated. This response does not appear to be related to age-related differences in ribosome biogenesis, since ribosomal mass accumulates similarly in Adult and Old muscle at 48 h. Rather injury, ER stress and/or impaired exercise-induced feeding potentiation are more likely to contribute to age-related anabolic resistance in late post-exercise recovery. We observe no induction of proteasome activity following acute resistance exercise and hypothesize that age-related reductions – not elevations – in skeletal muscle proteasome activity contribute to cellular dysfunction which may in turn contribute to sarcopenia.

## Author Contributions

DW, SB, and KB designed the study. DW and KB drafted the manuscript. All authors collected and analyzed the data, responsible for revising the intellectual content of the manuscript, and reading and approving the final version of the manuscript.

## Conflict of Interest Statement

The authors declare that the research was conducted in the absence of any commercial or financial relationships that could be construed as a potential conflict of interest.

## Abbreviations

BiP: endoplasmic reticulum binding protein
CHOP: CCAAT-enhancer-binding protein homologous protein
eIF2α: eukaryotic initation factor 2α
GAPDH: glyceraldehyde 3-phosphate dehydrogenase
IRS1: insulin receptor substrate 1
ITS-1: internal transcribed spacer 1
MPB: muscle protein breakdown
MPS: muscle protein synthesis
mTORC1: mechanistic/mammalian target of rapamycin complex 1
PDK: phosphoinositide-dependent protein kinase
PI3K: phosphoinositide 3-OH kinase
rpS6: ribosomal protein S6
TAF1B: TATA box binding protein-associated factor RNA polymerase I B
UBF: upstream binding factor.
